# Public Health Emergency and Crisis Management: Case Study of SARS-CoV-2 Outbreak

**DOI:** 10.3390/ijerph17113984

**Published:** 2020-06-04

**Authors:** Hemin Choi, Wonhyuk Cho, Min-Hyu Kim, Joon-Young Hur

**Affiliations:** 1 Graduate School of Public Administration, Seoul National University, Seoul 08826, Korea; hchoihm@gmail.com; 2 Wellington School of Business and Government, Victoria University of Wellington, Wellington 6011, New Zealand; wonhyuk.cho@vuw.ac.nz; 3 School of Public Affairs, Arizona State University, Phoenix, AZ 85004, USA; mkim218@asu.edu; 4 The Hainan University-Arizona State University Joint International Tourism College, Hainan University, Haikou 570004, China; 5 Korea Institute of Public Administration, Seoul 03367, Korea

**Keywords:** pandemic, public health, coronavirus, SARS-CoV-2

## Abstract

The SARS-CoV-2 pandemic has caused an unparalleled public health crisis, delivering an immense shock to humanity. With the virus’s health consequences largely unknown, different health systems around the globe have pursued various avenues of crisis management. South Korea, troubled early by the virus, was once the second most affected nation in the world. Arrays of measures in South Korea, such as large-scale diagnostic testing and technology-based comprehensive contact tracing, have brought about debates among public health experts and medical professionals. This case study describes the major cluster transmissions in SARS-CoV-2 hotspots in South Korea (such as a religious sect, a call center, logistics facilities, and nightclubs) and offers early observations on how South Korean public health authorities acted in response to the initial outbreak of the virus and to the new waves prompted by re-opening economies. We then discuss the way in which South Korea’s experience can act as a reference for shaping other countries’ public health strategies in pandemic crisis management.

## 1. Introduction

The SARS-CoV-2 outbreak is a public health emergency, and our globalized economy has helped spread this new infectious disease from Wuhan, China to every continent except Antarctica. South Korea was considered the second most affected nation until March 2020; however, as of late May 2020, the South Korean public health system is deemed a model of virus containment. South Korea’s public health authorities never implemented a “true” lockdown [1,2,3]. Most businesses stayed open, and borders have not been completely closed down to prevent overseas travel. This relative lack of mandate was due to two assumptions: (1) public health authorities have been confident about the country’s capacity to “trace, test, and treat” the SARS-CoV-2 patients, and (2) they assumed that citizens would generally follow social distancing rules without more restrictive measures. 

This case study describes the major cluster transmissions of SARS-CoV-2 in South Korea and analyzes the public health authorities’ emergency measures to the pandemic crisis. It follows the case-centric approach to case study, which is different from theory-centric or theory-building approaches [4], as it is used for a mechanistic explanation of the outcomes of a particular case. South Korea’s pandemic response is a complex case with a unique context: public health authorities have put in place various emergency measures without enacting stay-at-home orders or remaining in lockdown. Our analysis uses secondary sources, such as government archival data, public agencies’ publications, and media reports. 

This research asks how the South Korean public health system managed the pandemic crisis, flattened the curve without coercive measures like lockdown, and discusses the consequences. Then, we highlight important components of South Korea’s response to public health emergencies, with lessons for crisis management practice. Finally, the article identifies the distinctive features of South Korean public health authorities’ crisis management, such as widespread testing and diagnostic capacity, evidence informing and communication, contact tracing and alert texting, face mask distribution, and quarantine care. 

## 2. Recurring Waves of SARS-CoV-2: Clusters Involving a Religious Sect and Nightclubbers

**Figure 1:**
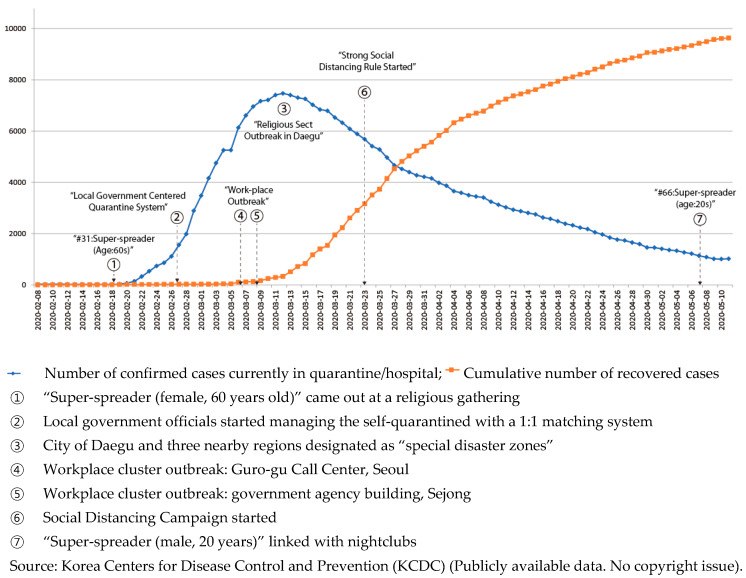
Number of quarantined and recovered patients.

Pneumonia patients with unknown etiology were first reported to WHO’s China Country Office in late December 2019. On 3 January 2020, Chinese national authorities identified 44 of these patients, and, on 20 January 2020, issued a statement reporting evidence of human-to-human transmission. The International Committee on Taxonomy of Viruses assigned the official name of “severe acute respiratory syndrome coronavirus 2” (hereafter SARS-CoV-2), which causes novel coronavirus disease 2019. While most patients with a SARS-CoV-2 infection have moderate symptoms, such as fever, coughing, or shortness of breath, a significant number of people have experienced severe, and sometimes deadly, pneumonia in both lungs. As of late May 2020, there were more than 5.8 million confirmed cases of SARS-CoV-2, with more than 360,000 deaths around the world. The WHO declared it a pandemic in March 2020, although experts argue that they should have warned of the danger earlier. The WHO, considered to have performed well during the 2002 severe acute respiratory syndrome epidemic, has reacted less effectively during the current outbreak, leading to calls for an overhaul of the entire organization. In South Korea, the outbreak got serious in late February 2020 and peaked in mid-March 2020, as shown in Figure 1.

In South Korea, the first confirmed case, identified on 20 January 2020, was a 35-year-old Chinese woman; the first confirmed case of a South Korean national with SARS-CoV-2 was identified on 23 January 2020. Like many other viruses, SARS-CoV-2 is an RNA virus, and the virus’s RNA sequence was released in late January 2020, to be used for producing diagnostic testing kits. In early February 2020, Korea’s Centers for Disease Control (Korea Centers for Disease Control and Prevention, hereafter Centers for Disease Control) authorized the first testing kits, made by a South Korean biotech company, to diagnose SARS-CoV-2 infection. Since then, the Centers for Disease Control have been aggressively expanding testing, alongside comprehensive contact tracing data and systematic quarantine care for infected people.

With these massive testing and contact tracing efforts by public health authorities, the virus initially seemed to be disappearing, as shown in Figure 1, averaging only about three new cases a day until mid-February. However, on 18 February 2020, a “super-spreader” [5], attended a gathering of a religious sect called the Shincheonji. This 61-year-old woman, known as “Patient 31,” was found to have transmitted SARS-CoV-2 to an unusually large number of people who attended religious events in the Shincheonji temple in the southeastern city of Daegu, home to 2.5 million people. Approximately three-quarters of the total number of SARS-CoV-2 cases ended up being clustered in Daegu, and, as of March 2020, about 60% of the total infections nationwide were traced to this religious group. Daily infections rose exponentially, nearing 1000.

The Shincheonji super-spreader developed a fever on 10 February 2020 but attended four Shincheonji events before being diagnosed with SARS-CoV-2. Public health authorities determined that this huge transmission was due to the behavioral characteristics of the religious group: members sit side-by-side in a cramped space for a significant amount of time during their temple service. This reinforced awareness of the need for social distancing, and the importance of following SARS-CoV-2 guidelines to avoid such places. Due to the reclusive and secretive nature of the religious sect, uncertainty in tracking escalated the outbreak. Group members, including “Patient 31,” tried to refuse diagnostic testing, thereby spreading the virus. While public health authorities have not found the precise epidemiological link between Patient 31 and her source of infection, further investigation by the Centers for Disease Control showed that group members traveled between South Korea and their Wuhan, China fringe branches in January 2020. 

This massive transmission left Daegu short of hospital beds and healthcare professionals. In March, the Centers for Disease Control announced a new guideline: treatment would depend on the severity of patients’ symptoms. Patients with mild symptoms were quarantined at community treatment centers, and only transferred to an intensive care unit or negative pressure room when their conditions deteriorated [6]. 

In March 2020, other clusters were reported outside of worst-hit Daegu. At River of Grace Community Church in Gyeonggi Province, more than 80 people tested positive. These cases drew international attention, as security camera footage showed church leaders spraying saltwater into followers’ mouths, as they believed that this practice would protect them from SARS-CoV-2. Another cluster outbreak with hundreds of new cases occurred at a Guro-gu call center in Seoul; this was especially concerning, as it happened in a workplace located in a commercial–residential mixed-use building, in a capital city with a population of 10 million. Additionally, in March 2020, an outbreak hit a central government complex located in Sejong city [7] and the oceans–fisheries ministry alone suffered more than 30 confirmed cases. 

**Figure 2:**
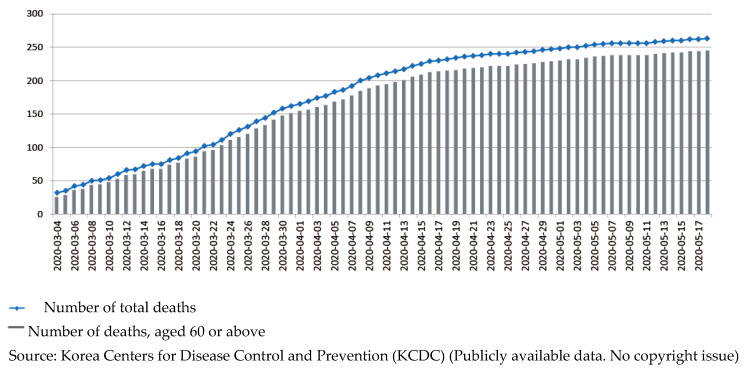
Cumulative number of deaths by SARS-CoV-2 in South Korea.

The public health authorities and the central and local headquarters for disaster and safety countermeasures exerted extensive measures to control the spread, and in April 2020, South Korea seemed to be recovered from large cluster transmissions of SARS-CoV-2. Death tolls seemed to flatten, as shown in Figure 2. In late April, when the number of new cases dropped to single digits for several days in a row, public health authorities decided to ease social distancing restrictions, shifting from strict measures (asking individuals and groups to cancel events, gatherings, and travels) to promoting “distancing in daily life.” People were able to go to restaurants, parks, and shops, although public health experts warned of the possibility of new clusters. 

National celebration over crushing the virus was short-lived. A 29-year-old man visited multiple nightclubs in a leisure district in Seoul on 1 May 2020 and tested positive for SARS-CoV-2 on 7 May 2020. These venues accounted for more than 160 cases and community transmissions. South Korea was under modified social distancing restrictions at the time. Clubs and shops were allowed to be open and receive customers, but people were still urged to avoid unnecessary contact with others and stay home.

**Figure 3:**
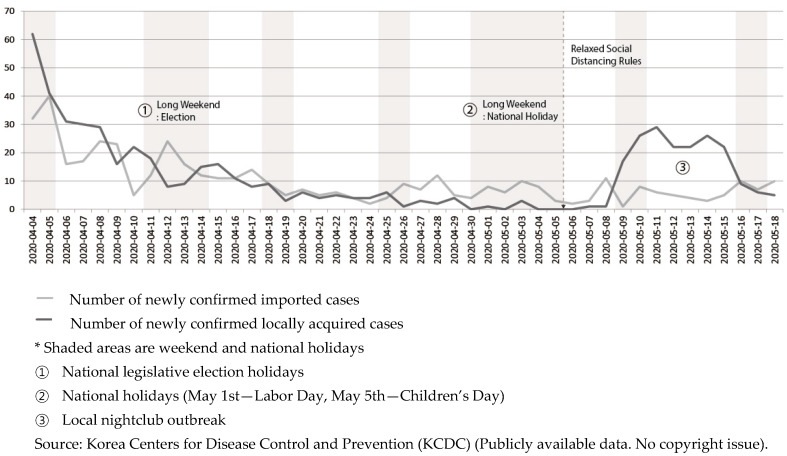
Trend of new confirmed cases.

After the nightclub cluster was discovered, nightclubs and bars were ordered to shut down indefinitely, which led to dismay among citizens who had hoped that life was returning to normal, based on the trend shown in Figure 3. Contact tracing was very tricky in this instance. Although clubs were supposed to verify all visitors’ names and contact details before allowing them to enter the venue, public health authorities found patron information insufficient for tracking, and sometimes completely bogus. Many of these nightclubs were popular among LGBT (lesbian, gay, bisexual, and transgender) communities in Seoul; fears of being involuntarily outed may have made some visitors hesitant to identify themselves. 

Another characteristic of SARS-CoV-2 transmission in South Korea is the disproportionate effect on younger people. In other nations, older people with underlying conditions were hit hardest. Table 1 presents data from the Centers for Disease Control, showing that the largest number of confirmed cases (27.70%) occurred in the 20–29 age group. This higher number of younger patients is a double-edged sword. Since the young are more likely to recover, the country suffers fewer deaths, as shown in Table 1. However, younger people, such as “Patient 66,” who visited five bars in one night and contacted as many as 2000 people, move around more. In addition, many young people remain asymptomatic, which complicates both testing and contact tracing.

In mid-May, the new waves of infections related to the nightclub clusters seemed to be under control, thanks to the public health system that South Korea used to get on the top of its earlier outbreaks. The daily confirmed cases had remained below 20, though there were still many reasons to be concerned about the newer waves, as shown in the new cluster in e-commerce logistics warehouses in late May—more than 90 new cases were linked to a cluster of infections at a logistics facility in Bucheon, west of Seoul, run by Coupang Corp.—Coupang is one of the largest e-commerce firms whose logistics facilities and warehouses scrambled to manage a surge in demand.

Taken overall, however, South Korea’s public health system has not been overwhelmed nationally while flattening the curve from new outbreaks, without stringent lockdown measures or other severe restrictions on movement, such as those implemented in New Zealand, Australia, England, and Canada—and many experts remain puzzled at the outcome in South Korea, particularly when considering the country’s proximity to China and large volume of travelers from there. In South Korea, while some businesses were restricted, and some people had to work from home or go on unpaid leave indefinitely, and most restaurants, shops, and leisure venues stayed open, and workplace habits have remained largely unchanged. Social distancing and the use of face masks were encouraged, but only on a voluntary basis, and as part of a public health campaign. New waves of clusters, such as the nightclub transmission or the more recent e-commerce warehouse outbreak, may be inevitable, as other cities or countries re-open their economies. However, under this “new normal” of recurring waves of infection, effective public health systems with adequate crisis management are crucial to establishing the balance between tight restrictions and restarting economic and social activities. 

## 3. Preparedness of the Public Health System: Resources and Human Capital

Hospitals and medical professionals form the core of mitigation and containment measures and are the heart of the response to the public health emergencies caused by the SARS-CoV-2 pandemic. Protecting these public health systems from being overwhelmed has been crucial. Exponential increases in numbers of confirmed cases of infection meant that many countries had to focus on the most vulnerable patients, particularly those who were older or suffered underlying medical conditions. It is easy to agree that those who are experiencing symptoms should have access to testing, but this is complicated by the availability of tests, their cost, and who is paying for them. 

Notably, diagnostic testing and treatment are free for confirmed patients of SARS-CoV-2 in South Korea, for both citizens and foreigners staying in the country. The South Korean public health authorities have maintained a solid commitment to free testing and free treatment principles, based on the Act on Infectious Disease Prevention. This free testing and treatment sparked public controversies, among both tax payers and policy makers, but the local governments and National Health Insurance Service (NHIC) agreed to continue covering all the costs, prioritizing risk aversion in order to rein in the transmission of the virus. Without the barrier of cost, residents in South Korea are more likely to voluntarily consult with public health authorities about their symptoms and contacts.

**Figure 4:**
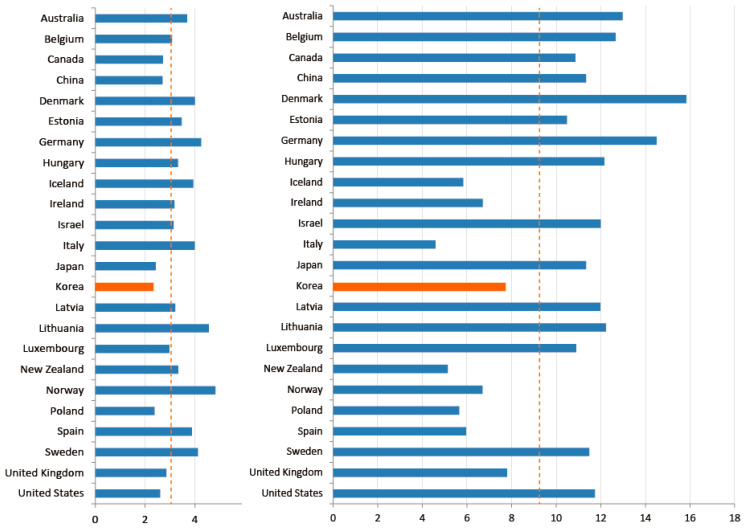
National health infrastructure of OECD countries. Note: Number of doctors/1000 inhabitants (left), number of nurses/1000 inhabitants (right). Source: OECD (Publicly available data. No copyright issue).

South Korea also experienced a “medical surge,” which refers to a heightened ability to provide adequate medical care during a crisis, exceeding the limits of the normal medical infrastructure. The early-stage Daegu outbreak was a definitional example of a medical surge. As shown in Figure 4, South Korea has among the lowest number of doctors per 1000 inhabitants (2.34); Denmark (4.00), Sweden (4.12), and Norway (4.82) have about twice as many doctors per capita. Numbers of nurses are slightly better. South Korea has 7.74 nurses per 1000 inhabitants, more than Italy (4.59), New Zealand (5.14), and Norway (6.7), but still lower than Denmark (15.84) and Germany (14.5). A common myth holds that South Korea, and Asian countries in general, enjoy greater human capital in the public health sector than others. In reality, however, South Korea’s health infrastructure is not necessarily better, and may be worse, than many other OECD member countries.

**Figure 5:**
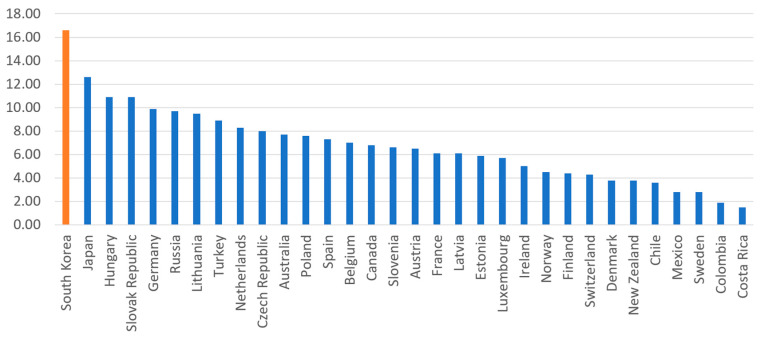
Number of consultations patients have with doctors in a year. Source: OECD (Publicly available data. No copyright issue).

While South Korea’s health infrastructure is below average among OECD countries, South Korea’s national health system offers care with low barriers of physical/geographical access to doctors, and at low cost. A universal national health care system covers almost all citizens, regardless of income level, at a standardized low cost [8,9]. Therefore, other than surgery, the price of medical treatment is almost the same as the so-called “Big Mac” price. Every administrative jurisdiction, furthermore, has its own local public health center, staffed with health care experts, including at least one full-time public doctor, also known as a public health physician (PHP). These certified physicians serve in a public health center or sub-center in a rural village, instead of serving in the military. This system allows (limited) medical resources to be reasonably allocated across all geographic locations of the country affordably. These low barriers to care explain why South Korea (16.6) far surpasses the OECD average (6.9) and the rest of the world in the number of consultations patients have with doctors in a year, as shown in Figure 5. The approachability and affordability of public health resources, not the sufficiency of them, may point to an important context of pandemic response. 

A significant number of PHPs are certified field epidemiologists, also known as Epidemic Intelligence Service (EIS) officers. During the early stage of the outbreak, these PHPs worked as local field epidemiologists, screening initial diagnostic test kits and promptly investigating the results. PHPs exercise various field-level discretions on contact tracing, based on an understanding of the patient’s situation, such as whether to coordinate with field epidemiologists from the headquarters to access a patient’s credit card and mobile phone usage, or to check security camera footage of the patient’s movements. These coordinated systems enabled the quick identification of the clusters, and allowed for swift follow-up decisions on the exercise and length of quarantine measures and the number of contacts to reach out to for testing.

Autonomic and Immuno-Vascular Mechanisms of Antihypertensive Effects of Taichi.

## 4. Findings: Results of Case Analysis

### 4.1. Widespread Testing and Diagnostic Capacity

The diagnostic capacity, or the ability to test a large number of patients, is of great importance, not only in identifying and treating the virus, but also in establishing the reliability of medical data on which to base follow-up decisions on the pandemic response. This capacity requires a healthy supply chain of testing kits, at both manufacturing and distribution levels, as well as an efficient testing system. Since the SARS-CoV-2 outbreak in January 2020, the South Korean public health system has tested more than 750,000 cases. Its daily capacity surpassed 15,000 tests very early in the response, in February 2020.

Just after the release of the RNA sequence of SARS-CoV-2, long before its first reported case, the South Korean public health authorities coordinated with the country’s reasonably well-developed biotech sector and started stashing testing kits. In January 2020, the drug safety ministry and the Centers for Disease Control swiftly authorized a pre-approval procedure, known as Emergency Use Authorization (EUA), for SARS-CoV-2 testing kits, to ease formalities and bureaucratic processes. Six days later, on 4 February 2020, public health authorities were able to use the authorized diagnostic kits for SARS-CoV-2 testing. 

All too often, red tape has worked as a barrier to building sufficient testing capacity amid public health crises [10]. However, the South Korean public health authorities effectively managed the tension between the compelling demand to provide the diagnostic kits for SARS-CoV-2 testing and the required level of quality assurance for rigorous diagnosis. South Korea decided to take pre-emptive state action, ensuring an abundant supply chain of testing kits and other necessary medical supplies, instead of waiting for the market mechanism to respond to demand. 

Contrary to its high responsiveness to the SARS-CoV-2 pandemic, South Korea’s management of the Middle East respiratory syndrome coronavirus (MERS-CoV) outbreak in 2015 failed the initial response to conduct pre-emptive quarantine and crisis communication [11]. Thanks to bureaucracies in the authorization process, a newly developed and faster in vitro kit for MERS-CoV testing was not approved for use. As a result, it took a minimum of several days to get confirmed results from a lab-based diagnosis. These lessons led to a law, passed in 2016, which allowed for the emergency use of testing kits, and expedited authorization procedures for in vitro diagnosis, in situations of significant public health crisis. 

Drive-through style testing stations, both time-efficient and safe, diffused from a public hospital (Kyungpook National University Hospital) to a local government (Goyang City), and then nationally and even internationally. Later, the phone booth-style testing facilities, also known as walk-through stations, were installed, adding the ease of installation and adjustment to the merits of speedy, safe testing. Whole sample collection procedures, which took about 30 min in previous screening stations, take only about five minutes in a walk-through system. Most of the excess time (more than 10 min) in the previous system was spent disinfecting the sample collection space in order to protect the next patient. The phone booth-style station allows medical personnel to examine patients from behind plastic-molded panels, eliminating the need for time-intensive cleanup. 

### 4.2. Tech-Powered Contact Tracing 

South Korea is known for its sophisticated mobile and digital technologies and transnational tech companies, and digital technology has been the core of aggressive “contact tracing” strategies. While confirmed patients were required to report symptoms and detailed contacts for previous days (even days when they did not present any symptoms), the public health authorities did not rely solely on this self-report. They used so-called digital surveillance technologies, which could be controversial, and various other sources of information, such as mobile phone data (including locational information of individuals), credit card statements, and security camera footage for tracing detailed contacts.

Public health authorities conducted in-depth investigations on SARS-CoV-2 hotspots, aimed at comprehensive contact tracing and follow-up measures encouraging quick testing. For example, during the outbreak at the Guro-gu call center in Seoul in March 2020, the Centers for Disease Control and local governments established a joint investigation team to perform epidemiologic analysis for contact tracing. This investigation traced detailed follow-ups for 14 days after the discovery of confirmed cases while the containment measures were in place. The call center was located in a 19-storey building in a highly populated urban area. Commercial offices occupied 11 floors and residential apartments took up the other eight floors. The public health experts closely tracked all patients under investigation (PUI), including 922 employees working in the commercial offices, 203 residents of the apartments, and 20 visitors. They determined each person’s detailed movements in the building, such as precise office seating and patterns of movement between floors, during the timeframe of 21 February to 8 March 2020. They tracked mobile phone location data and security camera footage in the building and sent more than 16,000 text messages to people traced who had spent more than five minutes in the building. The investigation team concluded from this in-depth contact tracing that, although both residents and employees in the building had regular contact in the elevators or the lobby, almost all of the patients were the ones who sat on one side of the 11th floor. This connection recognizes that the duration of close contact is an important risk factor of SARS-CoV-2 transmission, and that follow-up measures should consider it.

Digital surveillance technology was more heavily used in the recent outbreak in Seoul nightclubs, but the unique context of this cluster should be considered. The media reported that the nightclubs that the 29-year-old man visited were venues popular among LGBT communities, although those clubs do not openly advertise themselves as such, either on their official websites or on social media. That information, along with publicly released identifying details about the man’s age, movements, and place of residence, could mean that the man’s sexual orientation could be forcibly outed, which may risk his social and/or professional life. Even though these clubs were required to collect names and contact information and take temperatures before admitting guests, public health authorities’ contact tracing became trickier, as almost 2000 club patrons in the cluster left false or incomplete contact information or avoided follow-up calls. 

Tracing the nightclub cluster proved tougher than even the February outbreak clustered in the Shincheonji religious sect. Though these secretive group members were unwilling to cooperate with the public health authorities, the group at least had relatively comprehensive contact details of their 200,000 members. After some drama, the public health authorities obtained that detailed information and effectively traced them. However, tracking or tracing the nightclub-goers required very different approaches. 

The public health authorities and local governments employed a more technology-based approach to comb through the nightclub-goers. They requested that all mobile network operators (LG, SK, and KT) submit signal tower records, and found that 10,905 people were in the proximity of the nightlife suburb of Seoul from 24 April to 6 May 2020. The public health authorities decided to send text messages to all of them, as an all-out effort to reach people potentially exposed to the virus, asking recipients to get tested and self-quarantine. The cell tower information is very detailed: in the nightclub area, there are signal towers every 50 to 100 m. The public health authorities narrowed the tracing down to people whose phone got the signal from the cell tower for more than 30 min (cell phones did not have to be used to be traced, just powered on), while maintaining the ability to expand the tracing coverage to people in the area for a shorter time. The public health authorities and police forces investigated all security camera footage, both in nightclubs and on the street, tracking the movements from the bars to the subway stations. There are more than 37,000 public security cameras managed by Seoul city (and more than 1,150,000 public security cameras in the country), and the police force can request to investigate privately installed security camera footage; surveillance technology, if authorized, is able to trace almost every movement. The public health authorities traced credit card transactions of more than 500 people to complement the mobile data. 

The use of digital surveillance technology for contact tracing exposed the delicate balance between privacy issues and public health emergency management. South Korean public health authorities’ effective containment of the SARS-CoV-2 has required the public to tolerate a certain degree of privacy infringement; authorities can trace people’s movements through digital technology, mobile phone data, and security camera footage with or without their consent. We should note that this condition of tolerance among South Koreans cannot be easily generalized to other contexts. Even in South Korean society, where in modern history citizens often had to cede control over their private information to government authorities, this aggressive system of contact tracing sparked fears and dissent, particularly among minorities, such as religious sects, foreigners, and LGBT people. These text messages, indicating precise knowledge of one’s whereabouts at a specific day and time, were understandably ill-received, as was the knowledge of credit card monitoring. 

### 4.3. Public Communication and Alert Texting

The failure of public health officials to inform citizens can lower confidence in the public response to the pandemic, which in turn hinders the effectiveness of voluntary measures, such as social distancing. For this reason, communication with citizens is particularly critical for democracies [12], in which many of the pandemic responses are voluntary and do not rely on coercive authoritarian state orders [13,14,15]. In South Korea, the Centers for Disease Control have communicated relevant information to citizens and policy makers through various channels. 

The Centers for Disease Control have held media briefings since the first confirmed case was reported. One noticeable characteristic of these press conferences has been that every detail of the shared materials in the briefings is identical to those in ministerial debriefings. This means that citizens enjoyed high levels of understanding and could make informed decisions on circumstances such as self-quarantine or face masks. Presenters were very engaged with journalists and the general audience, even when communicating technical details, demonstrating the media training of public health specialists. 

The public health authorities played the role of fact-checkers in order to prevent infodemics about the virus and promoted social distancing measures through various media outlets. They offer real-time updates on the spread of the virus via social media, websites, and television, to reach out to the largest possible population. Open data platforms provided details of testing and contact tracing, so that programmers and app developers could innovate effective and efficient ways to provide alternatives. 

Almost every movement taken by confirmed patients is released in real time, and all people with a registered mobile phone get text alerts when there is SARS-CoV-2 hotspot nearby. Everyone gets automatic emergency messages, such as “The 15th imported case tested positive in Jongno-gu district is staying at the Somerset Palace Hotel in Seoul. For more information about the paths, such as restaurants and shops traced, please visit our website.” Alert texting details exhaustive lists of routes with the estimated times of visits. The public authorities send texts within 24 h after any case is confirmed test-positive. 

The system of emergency text alerts creates a trade-off between publicizing information about confirmed cases and protecting patient privacy. Such highly detailed information about the movements of each known case, sometimes including age, address, and employer, can reveal a patient’s identity. Various stigmas, such as the outing concerns of nightclub-goers, may prevent people from coming forward for testing. To respond to these concerns, the public health authorities announced the adoption of “anonymous testing” options on 14 May 2020, publicly promising to collect phone numbers and not names. Soon after the announcement, the number of those who came forward for testing had doubled, to more than 35,000 tests, from nightclub-related clusters. The authorities have asked the public not to blame specific individuals or communities and warn that prejudiced speech on confirmed cases will worsen the pandemic, as some of the clubbers may lie low to avoid demonization. South Korea’s journalist associations drafted and released pandemic reporting guidelines, requesting that the media avoid using sensational language or infringing on patients’ privacy. South Korean public health authorities have clearly communicated that it is a criminal offense to leak the private information of SARS-CoV-2 patients. 

### 4.4. Face Mask Rationing through Integrated Health Data and Pharmacy Inventory Apps

The South Korean health authorities recommend wearing a face mask covering the nose and mouth, to protect other people from SARS-CoV-2 infection, based on research showing that face masks reduce airborne virus transmission more than threefold. The health outcomes for the wearer have been controversial; scientific studies have been inconclusive on its effectiveness. However, public health experts generally agree that face masks provide at least a “positive externality” in reining in community transmission, since they reduce the chances that people will infect others nearby [16]. Indeed, many countries that initially discouraged wearing face masks later turned it back, asking citizens to cover up their noses and mouths, even with make-shift masks, whenever out in public. In South Korea, this recommendation was not too disruptive, as most average citizens were already accustomed to wearing filtration face masks due to worsening air quality. Even though the SARS-CoV-2 curve seems to be flattened, people still continue to wear masks to protect themselves.

A more problematic issue was the shortage of face masks, particularly the filtration masks (such as N95 or KF-94 particulate-filtering facepiece respirators) needed by health professionals. South Korean public health authorities and state economists shared the view that face masks, hand sanitizers, and other core medical supplies are different from many other goods in the market, as they benefit more than just the person using them. As such, the government decided to impose price controls and rationing to respond to mask shortages. They worried that industries, overwhelmed by demand, would respond to those willing to pay higher prices, even if these customers were panic-buying, hoarding, or reselling the supplies at even higher prices.

South Korean public health authorities limited the price of a face mask to about $1.20, which was slightly below the market price at the time. They also imposed rationing of two face masks per week per person and prioritized health professionals dealing with SARS-CoV-2 infections. The authorities also bought a large share of the available masks, to distribute them at a low price to people located in SARS-CoV-2 hotspots. They were able to efficiently monitor these prices and restrictions thanks to the digitalized pharmacy health data portal run by the Health Insurance Review and Assessment Service (HIRA) platform. Through this integrated platform, pharmacists and public health authorities can review whether a customer has already bought a face mask elsewhere before a sale.

The public health authorities divided citizens into five groups, based on date of birth, and assigned each group to a specific day of the week to buy face masks. They required every citizen to present identification cards, allowing pharmacists to review their mask purchasing history. They also asked each pharmacy to record every mask transaction in the HIRA data platform with a person’s ID number. Furthermore, the authorities required pharmacies to digitally update stock and sales in the data system and asked them to release the data to the public, allowing developers to come up with apps showing real-time mask stock levels at each pharmacy. Participation in data release was not mandatory, but 22,000 pharmacies out of 23,000 nationwide (95%) have agreed to share it. The nation’s largest chat app, KakaoTalk, and Naver, its largest search engine, began providing map services showing a pharmacy’s inventory of face masks in real time. Many other similar apps followed.

In terms of production, the authorities banned the export of masks until local production was sufficiently ramped up to distribute the masks in the rationed market. At the early stage of market intervention, authorities failed to supply the promised number of masks, and pharmacies did not have enough stock for all customers, as stipulated under the adopted rationing scheme. Many citizens were frustrated, and this sparked public criticism. But soon production stabilized, and the average citizen could buy masks without waiting in a long queue. As of May 2020, the authorities announced that over 80 percent of the demand for masks rationed in the market was supplied by local production.

### 4.5. Two Weeks’ Quarantine Care: From Physical Health Conditions to Psychological Health Conditions

The South Korean public health authorities approached quarantine measures as comprehensive care, rather than just limiting movements or contacts, with the aim of high levels of compliance. When inbound travelers arrived at the airport, medical personnel asked them to install a self-health check app to continuously monitor them. They also escorted people outside of the building for testing if needed; either they or local governments boarded them on a chartered minivan or a bus to a police facility to await test results overnight. People without symptoms, or from less risky regions, were allowed to go directly to quarantine facilities either provided by the authorities or by themselves [17]. In most facilities provided by local governments, all food and amenities were offered in the form of a “care box,” and people could usually see their test results the following morning. Passengers with SARS-CoV-2 symptoms, or those who have traveled to high-risk regions, were placed in quarantine. All in-bound travelers were required to submit their health conditions on the self-health check app, called the “Self-Quarantine Safety Protection Mobile App,” during their 14-day incubation period.

During the 14 days, people received home-quarantine guidance, and information from their local government about how to report symptoms they may have developed. Local government officials assigned to self-quarantined patients called two times a day, not only checking the physical health condition, but also monitoring their psychological health. If necessary, psychological counseling services were also provided, not only for quarantined patients, but also for family members who are in the home, since psychological condition strongly linked with fundamental vulnerability [18,19]. The Korean Neuropsychiatric Association, with the Ministry of Health and Welfare, has published guidelines to help quarantined people deal with anxiety, encouraging frequent online communication. South Korea’s ICT-based quarantine care system was made possible by its superlative penetration rate in terms of smartphone ownership and internet usage. To this end, technology plays an important role in terms of citizens’ quarantine quality of life; it prevents isolation and supports close care by the government.

Another quarantine option was a “Living and Treatment Center (Community Treatment Center),” which is an isolation facility for confirmed patients with mild symptoms. This service was provided to patients with old parents or young children, without extra room at home for self-quarantine. Depending on their capabilities, each local government provided a slightly different quarantine care package and living facility options. Some cities provided a full package of medical equipment, individual relief kits, and hygiene kits. Some cities offered living facilities without cost, and others charged.

## 5. Discussion 

The pandemic is still overwhelming many other countries’ healthcare systems with exponentially rising numbers of patients, creating unprecedented challenges. South Korean public health authorities deployed extensive digital surveillance technologies in fighting SARS-CoV-2, and contact traced huge numbers of potential or probable cases, to test and quarantine them before further community transmission. The case of South Korea also shows that it is important to provide sufficient information to the public on SARS-CoV-2 cases, so that citizens may comply with public health measures, such as wearing face masks and social distancing. As to South Korea’s approach to face mask distribution, economists, though skeptical about price controls, may acknowledge that this case is an allowable rare exception. Though price controls are generally inferior to market forces, the market sometimes needs a bit of help [20], and face mask rationing in South Korea may be the case that proves it.

Many countries had to implement restrictive lockdowns, affecting their whole population and economy, while South Korea took a different approach: let transmission happen and then quickly control it. The South Korean public health authorities’ approach was not preventive but reactive. New waves recurred in an open economy and across relatively open borders. A significant number of people died of the SARS-CoV-2 infection; approximately 260 deaths may seem small compared to the tolls in other countries, but the number is large in the epidemic history of the country.

New waves of clusters, such as those of the Shincheonji sect or the nightclubs, could have been avoided if public health authorities had implemented more preventive measures, such as nation-wide lockdown or border closings, such as that in New Zealand. There could have been many “what if” moments, really. But “what if” this pandemic does not easily end in a short period of time, and economies must re-open? No public health system can lock down all activity for an indefinite timeframe. Therefore, public health systems should build more capacity, in order to effectively deal with unpredictable new waves of outbreaks.

What is most notable in South Korea’s pandemic response is the success of these measures in controlling transmission; this capacity was largely attributable to the MERS experience in 2015. Citizens’ tolerance to digital surveillance technology and privacy infringement in exchange for a lockdown-free economy is another unique condition, not easily generalized or benchmarked. However, public health experts may want to consider the use of digital surveillance. A deliberate analysis on its costs and benefits may illuminate a path out of this unprecedented, and possibly unending, global crisis.

## 6. Conclusions

This article is a very early observation and presents only a very incomplete picture. At the time of writing, the pandemic may not have reached its peak. More serious waves may occur in the future. More follow-up research is needed, with in-depth analysis on each case, to understand and control the virus.

New waves of clusters, such as those of the Shincheonji sect or the nightclubs or e-commerce warehouses, could have been avoided if public health authorities had implemented more preventive measures, such as nation-wide lockdown or border closings, such as that in New Zealand. There could have been many “what if” moments, really. But “what if” this pandemic does not easily end in a short period of time, and economies must re-open? No public health system can lock down all activity for an indefinite timeframe. Therefore, public health systems should build more capacity, in order to effectively deal with unpredictable new waves of outbreaks.

## Figures and Tables

**Table 1:** Total cases by age as of 29 May 2020.

Age Group	Confirmed Cases (%)	Deceased (%)	Mortality Rate (%)
80+	495 (4.34)	131 (48.70)	(26.46)
70–79	724 (6.35)	79 (29.37)	(10.91)
60–69	1400 (12.28)	39 (14.50)	(2.79)
50–59	2023 (17.74)	15 (5.58)	(0.74)
40–49	1513 (13.27)	3 (1.12)	(0.20)
30–39	1283 (11.25)	2 (0.74)	(0.16)
20–29	3158 (27.70)	0 (0.00)	-
10–19	650 (5.70)	0 (0.00)	-
0–9	156 (1.37)	0 (0.00)	-
total	11,402 (100)	269 (100)	-

Source: Korea Centers for Disease Control and Prevention (KCDC) (Publicly available data. No copyright issue).
